# Myxomavirus Serp-1 Protein Ameliorates Inflammation in a Mouse Model of Duchenne Muscular Dystrophy

**DOI:** 10.3390/biomedicines10051154

**Published:** 2022-05-17

**Authors:** Alexander B. Andre, Liqiang Zhang, Jalen D. Nix, Nora Elmadbouly, Alexandra R. Lucas, Jeanne Wilson-Rawls, Alan Rawls

**Affiliations:** 1 Molecular and Cellular Biology Graduate Program, School of Life Sciences, Arizona State University, Tempe, AZ 85287, USA; abandre@asu.edu (A.B.A.); jdnix@asu.edu (J.D.N.); jeanne.wilson-rawls@asu.edu (J.W.-R.); 2 Biodesign Center for Personalized Diagnostics, Biodesign Institute, Arizona State University, Tempe, AZ 85287, USA; liqiang.zhang@asu.edu (L.Z.); arlucas5@asu.edu (A.R.L.); 3 Center for Immunotherapy, Vaccines, and Virotherapy, Biodesign Institute, Arizona State University, Tempe, AZ 85287, USA; nelmadbo@asu.edu

**Keywords:** Duchenne muscular dystrophy, serpin, fibrosis, inflammation, *Myxoma virus*

## Abstract

Duchenne muscular dystrophy is an X-linked disease afflicting 1 in 3500 males that is characterized by muscle weakness and wasting during early childhood, and loss of ambulation and death by early adulthood. Chronic inflammation due to myofiber instability leads to fibrosis, which is a primary cause of loss of ambulation and cardiorespiratory insufficiency. Current standard of care focuses on reducing inflammation with corticosteroids, which have serious adverse effects. It is imperative to identify alternate immunosuppressants as treatments to reduce fibrosis and mortality. Serp-1, a Myxoma virus-derived 55 kDa secreted glycoprotein, has proven efficacy in a range of animal models of acute inflammation, and its safety and efficacy has been shown in a clinical trial. In this initial study, we examined whether pegylated Serp-1 (PEGSerp-1) treatment would ameliorate chronic inflammation in a mouse model for Duchenne muscular dystrophy. Our data revealed a significant reduction in diaphragm fibrosis and increased myofiber diameter, and significantly decreased pro-inflammatory M1 macrophage infiltration. The M2a macrophage and overall T cell populations showed no change. These data demonstrate that treatment with this new class of poxvirus-derived immune-modulating serpin has potential as a therapeutic approach designed to ameliorate DMD pathology and facilitate muscle regeneration.

## 1. Introduction

Duchenne muscular dystrophy (DMD) is an X-linked disease afflicting 1 in 3500 males, and is characterized by muscle weakness and wasting during early childhood, with a severe loss of ambulation and death by early adulthood [1]. Significant cardiac complications begin to be detected in boys at 9–10 years of age, eventually causing severe systolic and diastolic dysfunction. Approximately 90% of DMD patients experience myocardial cell death and dilated cardiomyopathy, with associated increases in mortality [2]. In both skeletal and cardiac muscle, functional loss is linked to chronic inflammation and the accumulation of endomysial fibrosis [3,4]. Despite identifying mutations in the dystrophin locus as the genetic basis for DMD, there is still no curative treatment [5]. Thus, the development of therapeutic approaches that mitigate fibrosis remains imperative for improving the quality of life for these individuals.

Skeletal muscle possesses an intrinsic ability to repair acute injuries dependent on signaling crosstalk between the myogenic cells and the innate immune system [6,7]. Chemokines and T helper type 1 (Th1) cytokines that activate and recruit the satellite cells necessary for new muscle formation also recruit the pro-inflammatory polymorphonuclear cells and M1 macrophages. In mice, M1 macrophage infiltration peaks 3 days post-injury; these cells clear necrotic muscle fibers by phagocytosis and the release of reactive oxygen species and inflammatory cytokines, such as interferon-γ (IFNγ) and tumor necrosis factor-α (TNFα) [8,9]. This phase of the repair process then transitions to an anti-inflammatory response that promotes skeletal muscle differentiation and maturation, and inhibition of myolysis, through the release of T helper type 2 (Th2) cytokines. These changes coincide with the infiltration and polarization of macrophages of the pro-regenerative M2 phenotype, which peak at 7 days post-injury. The M2 macrophages secrete cytokines, such as interleukin-10 (IL-10) and transforming growth factor β (TGFβ), to aid in the resolution of inflammation and induce the differentiation of satellite cells [10,11].

In individuals with DMD, the loss of the dystrophin protein and the decoupling of the force-transmitting costameric cytoskeleton disrupts the sarcolemmal membrane integrity [12]. This results in constant repeated asynchronous rounds of muscle injury, causing chronic inflammation and disrupting the balance of M1 and M2 macrophages, leading to the recruitment of an alternate M2 macrophage subtype that expresses high levels of TGFβ and fibronectin [13,14,15]. TGFβ, in turn, promotes fibrosis through excessive collagen deposition from activated resident fibroblasts [16,17] and differentiated fibro–adipogenic progenitor cells (FAPs) [18]. Chronic inflammation also disrupts the expression of the pro-regenerative cytokines required for muscle fiber differentiation and maturation. Consequently, this results in the disruption of efficient skeletal muscle repair and reduced contractility.

The fibrinolytic/plasmin cascade is another important pathway for regulating inflammation and extracellular matrix (ECM) remodeling during muscle repair [19,20,21]. Urokinase-type plasminogen activator (uPA), a key component of the pathway, coordinates a plasmin-mediated proteolytic cascade when bound to its receptor (uPAR). This uPA/uPAR complex activates matrix metalloproteinases (MMPs) that are essential for ECM breakdown, activation of TGFβ and IGF-1, and inflammatory macrophage invasion at sites of tissue injury [22]. Furthermore, the uPA/uPAR complex binds to integrins at the cell surface to directly coordinate cell migration. uPA activity can be inhibited by the product of the serine proteinase inhibitor SERPINE1 gene, known as plasminogen activator inhibitor-1 (PAI-1) [23,24], through mechanisms that include serine protease inhibitory function, increased endocytosis, blocking activation of matrix metalloproteinases (MMPs), and reducing immune cell invasion [25,26]. TGFβ has been shown to promote the expression of PAI-1, suggesting the presence of an autocrine loop with uPA [27].

In mouse studies of cardiotoxin-induced acute muscle injury, uPA activity rapidly increased and was critical for pro-inflammatory macrophage and T cell infiltration and muscle regeneration [28,29]. In contrast, PAI-1^−/−^ mice demonstrated enhanced skeletal muscle repair in response to acute injury [20,28], consistent with PAI-1 inhibition of acute muscle regeneration. Interestingly, the uPA/uPAR signaling pathway appears to exacerbate fibrosis under chronic inflammatory conditions associated with DMD [27]. Using *DMD^mdx^* (mdx) mice that recapitulate DMD pathology in the aging process, inactivation of PAI-1 led to increased fibrosis, which can be ameliorated by the inactivation of uPA. It has been proposed that disruption of the uPA/PAI-1 autocrine loop via the overexpression of TGFβ in resident fibroblasts is the primary cause of fibrosis. This indicates that uPA/uPAR, as well as PAI-1, may provide potential therapeutic target pathways for DMD.

The current standard of care for DMD patients is corticosteroid therapy, such as treatment with prednisone/prednisolone [30]. These drugs have potent anti-inflammatory effects, but there are severe side effects, such as excessive weight gain, diabetes, cataracts, behavioral changes, delayed growth, and osteoporosis, that can contraindicate use [31]. In addition, long-term use of glucocorticoids is associated with skeletal muscle atrophy [32,33,34]. More recently, deflazacort, a derivative of prednisolone, has been approved for DMD; it has similar efficacy to prednisone with fewer side effects, but is associated with behavior changes and cataracts. Furthermore, data on the role of deflazacort in bone fractures and growth delay are not consistent [31]. The cardiac effects of corticosteroids are poorly studied. Some reports indicate improved cardiac function and delayed onset of cardiomyopathy [35,36,37]. However, it was also demonstrated that beginning treatment in younger DMD patients actually led to premature cardiomyopathy development [38].

There is an unmet need for pharmacological agents that inhibit inflammation without the adverse side effects of corticosteroids for DMD patients. In this study, we describe the evaluation of a new class of immune-modulating therapeutic for treating DMD that targets both inflammation and fibrosis. The Myxoma virus-derived serpin, Serp-1, binds and inhibits uPA/uPAR in activated macrophages, and has some shared protease targets with PAI-1 [39]. Treatment with purified native Serp-1 has demonstrated both acute and long-term efficacy in modulating inflammation in a wide range of inflammatory disorders and injuries, including atherosclerosis, transplant, wound healing, and spinal cord injury [39,40,41,42]. More recently, a modified Serp-1 protein, PEGSerp-1, with a longer half-life (~8 h), was shown to reduce inflammation and fibrosis in healing corneal wounds, and reduced macrophage invasion of alveoli in a mouse model of diffuse alveolar hemorrhage [43,44,45]. Based on these previous studies, we examined whether the pegylated version of the viral Serp-1 protein, PEGSerp-1, would ameliorate the chronic inflammatory pathology of DMD. Using mice deficient in dystrophin and the related utrophin gene (*DMD^mdx^ /Utrn^−/−^*), which recapitulates the juvenile pathology of DMD [46,47], we observed that PEGSerp-1 reduced diaphragm fibrosis after 28 days of treatment. This effect was associated with reduced pro-inflammatory M1 macrophage infiltration and improved skeletal muscle repair.

## 2. Materials and Methods

### 2.1. Protein Production and Purification

Serp-1 (m008.1L; NCBI Gene ID# 932146) was expressed in a Chinese hamster ovary (CHO) cell line (Viron Therapeutics Inc., London, Ontario, CA, USA). The Serp-1 protein used in this research is GMP-compliant and purified by continuous chromatographic separation. The purity of Serp-1 is >95%, as determined by Coomassie-stained SDS-PAGE and reverse-phase HPLC. Serp-1 was endotoxin-free, as detected by limulus amebocyte lysate (LAL) assay. Serp-1 was incubated with mPEG-NHS (5 K) (Nanocs Inc., #PG1-SC-5k-1, New York, NY, USA) in PBS (pH 7.8) at 4 °C overnight to modify the protein according to standard PEGylation protocols [43]. PEGylated-Serp-1 was purified by FPLC using an ÄKTA pure protein purification system with Superdex-200 [43].

### 2.2. PEGSerp-1 Treatment

PEGSerp-1 (*n* = 5 mice), or a saline control (*n* = 4 mice), was administered to *DMD^mdx^ /Utrn^−/−^* mice by intraperitoneal injection (ip.) daily for 28 days, at 100 ng/gm body weight in 100 μL saline or 100 μL saline only, beginning at 4 weeks of age. Similarly, a preliminary study was conducted using DKO mice treated with native Serp-1 (*n* = 3) at 100 ng/gm body weight or saline (*n* = 3).

### 2.3. Mice

*DMD^mdx^/Utrn^−/−^* (DKO) mice were purchased from Jackson Laboratory (Bar Harbor, ME, USA) and bred and housed in a vivarium at Arizona State University (ASU). For the fibrosis measurements only, muscle from C57BL/10J (WT) mice (*n* = 3), which is the genetic background of the DKO mice, was used. All mice were kept on a 10 h light:14 h dark schedule with *ad libitum* access to food and water. ASU is accredited by the Association for Assessment and Accreditation of Laboratory Animal Care (AALAC). All procedures were carried out in compliance with the ASU Institutional Animal Care and Use Committee (IACUC) and AALAC under an approved research protocol.

### 2.4. Histology and Immunohistochemistry

Diaphragm skeletal muscle was dissected from euthanized mice and fixed overnight at 4 °C in 4% paraformaldehyde and embedded in paraffin. For Mason’s trichrome staining, 5 μm sections were dewaxed in xylenes and rehydrated through graded alcohols and then post-fixed in Bouin’s fixative (Sigma-Aldrich, St. Louis, MO, USA). Sections were stained in modified Wiegert’s iron hematoxylin (Sigma-Aldrich), and then color differentiation was performed in acid alcohol followed by a trichrome stain. Sections were dehydrated through graded alcohols to xylenes, mounted, and immunohistochemistry (IHC) was performed. Sections were stained for CD3 (Abcam, 5690; 1:100, Cambridge, MA, USA), iNOS, Abcam 15,323; 1:100), and arginase-1 (Arg1) (Cell Signaling, 93,668, 1:200, Danvers, MA, USA). HRP-conjugated goat anti-rabbit IgG secondary antibodies were applied at 1:500 for 1 h. Only the HRP-conjugated secondary antibody, without a primary antibody, was used as a negative control for each stain. Antigens were revealed with ImmPACT DAB (Vector Labs, San Francisco, CA, USA), counterstained with Gil’s formula #3 Hematoxylin and mounted with Cytoseal XY. Random fields were imaged on each section using CellSens software and an Olympus BX50 microscope. The data are expressed as the average number of positive cells per 40× field. All counts were performed by blinded evaluators.

### 2.5. Fiber Diameter

The minimal Ferret’s diameter of muscle fibers containing centralized nuclei was manually measured on transverse Masson’s trichrome-stained sections using ImageJ software. Average fiber diameters were calculated for each treatment. The distribution of fiber diameters was determined by pooling into 4 μm increments, and are presented as percent of total fibers. All measurements were performed by a blinded evaluator.

### 2.6. Statistical Analysis

Data were analyzed in R by one-way ANOVA followed by post hoc Tukey tests.

## 3. Results

### 3.1. PEGSerp-1 Decreases Skeletal Muscle Fibrosis

Progressive fibrosis of the skeletal and cardiac muscles is a major contributor to morbidity and mortality in DMD patients. The mdx mice commonly used to study DMD pathology, display modest symptomology until 6 or more months of age [48], which is inconsistent with the pediatric human disease. Thus, we chose to use *DMD^mdx^/Utrn^−/−^* double knockout (DKO) mice for these studies [46,47]. Using DKO mice to model the progression of human DMD, there is onset of symptoms at 4–6 weeks of age, with progressive loss of ambulation, increased severity of kyphosis, and weight loss. By 10–12 weeks, they exhibit respiratory insufficiency, loss of hindlimb use, and severe weight loss; most are unable to survive past 12 weeks of age [46,47].

**Figure 1:**
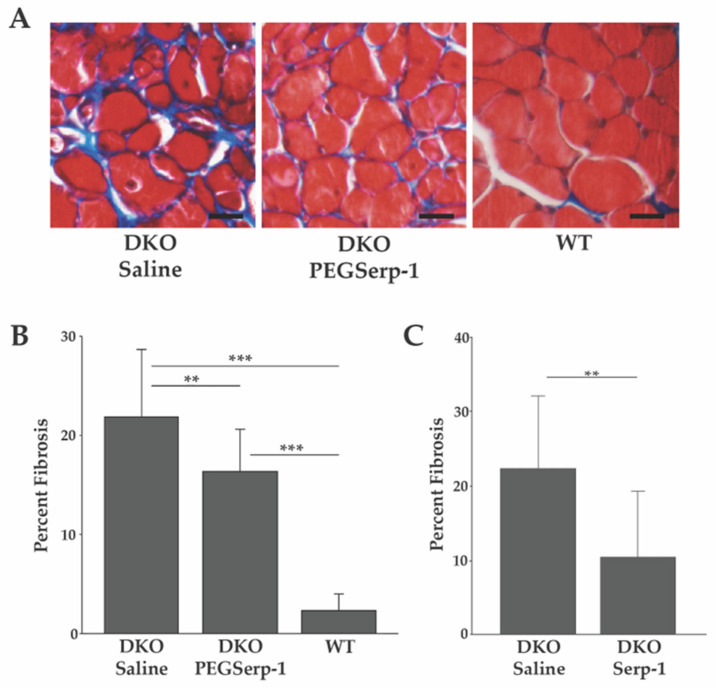
PEGSerp-1 treatment reduces skeletal muscle fibrosis. (**A**) Diaphragms stained with Masson’s trichrome. Note the collagen deposition (blue). (**B**) Fibrosis was measured as the average percent area per field ± s.d. using ImageJ (*n* = 29 fields per treatment group). (**C**) DKO mice treated with native Serp-1 protein or saline were similarly analyzed. PEGSerp-1/Serp-1-treated diaphragms had decreased fibrosis. ** *p* < 0.01, *** *p* < 0.001. Scale bars = 10 µm.

We assessed the potential of PEGSerp-1 to ameliorate the chronic inflammatory environment and subsequent development of fibrosis in DKO skeletal muscle. Four-week-old DKO mice were administered PEGSerp-1 (*n* = 5), or a saline control (*n* = 4), daily for 28 days. Deposition of endomysial collagen in the diaphragm muscle was used as a measure of the extent of fibrosis in PEGSerp-1- and saline-treated mice. Collagen was visualized by Masson’s trichrome staining of histological transverse sections (Figure 1A). An initial study, in which DKO mice were treated with the unmodified Serp-1 protein, demonstrated reduced fibrosis in the diaphragm (Figure 1C). These diaphragms had an average fibrotic area of 10.61% ± 8.92% s.d., as compared with 22.55% ± 9.77% s.d., for saline-treated controls, *p* < 0.01 (Figure 1C). Interestingly, PEGSerp-1-treated DKO diaphragms showed significantly less fibrosis than those treated with saline, with an average area of fibrosis of 16.32% ± 4.31% s.d., *p* < 0.01 (Figure 1A,B). Saline-treated DKO diaphragms demonstrated significant levels of fibrosis, with an average fibrotic area of 21.87% ± 6.8% s.d. (Figure 1A,B). PEGSerp-1- and saline-treated DKO diaphragms demonstrated significantly more fibrosis than WT, *p* < 0.001. Untreated wild type (WT, *n* = 3) animals, as expected, had much less fibrosis, with an average percent area of 2.29% ± 1.68%s.d., *p* < 0.001 (Figure 1A,B). Thus, PEGSerp-1 treatment reduced fibrosis in DKO skeletal muscle.

### 3.2. PEGSerp-1 Treatment Increased Fiber Diameter of DMD Muscle

Fibrosis is associated with the loss of skeletal muscle function, and it affects the growth and maturation of repaired muscle fibers [49]. Thus, the diameter of regenerated myofibers was used as a measure of the success of the repair process. As above, 4-week-old DKO mice were administered PEGSerp-1 or a saline control daily for 28 days. The Ferret’s minimal diameter of myofibers was measured in histological sections for all fibers with centrally located nuclei, a hallmark of fiber repair.

**Figure 2:**
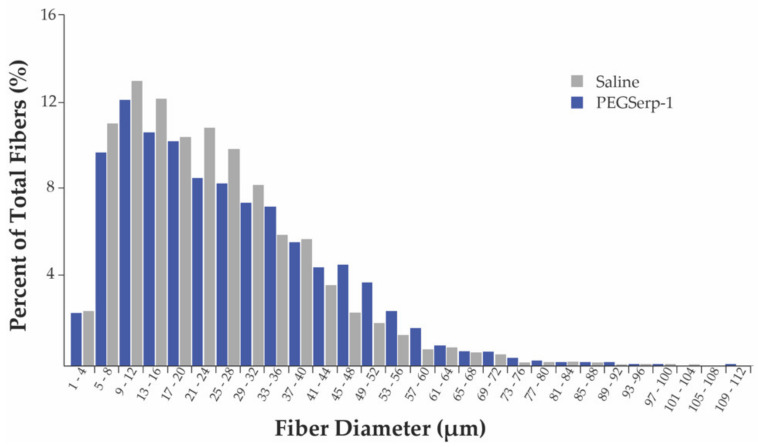
Distribution of Ferret’s minimal diameters of skeletal muscle fibers. Diaphragms from DKO mice treated with PEGSerp–1 or saline were stained with Masson’s trichrome. Images were taken at 40× and diameters measured. Data are presented as a percentage of total fibers counted. PEGSerp-1 treatment increased the percentage of myofibers with diameters of 33–64 μm, and significantly increased overall average fiber diameter as compared to saline-treated muscle, *p* < 0.001.

Interestingly, PEGSerp-1-treated DKO muscle had an increased percentage of fibers with diameters between 33–64 μm (Figure 2). The overall average diameter of regenerated myofibers in the PEGSerp-1-treated DKO diaphragms was 25.99 μm ± 16.00 s.d. (*n* = 3693), significantly larger than that of the saline-treated DKO muscle, 23.30 μm ± 14.86 s.d. (*n* = 3479), *p* < 0.001. These data are consistent with a preliminary study using with unmodified Serp-1, which demonstrated an increased percentage of myofibers with diameters of 13–36 μm (Supplementary Material Figure S1). These data indicate that PEGSerp-1 treatment resulted in significantly increased myofiber diameter in DKO muscle.

### 3.3. PEGSerp-1 Reduced Pro-Inflammatory M1 Macrophage Numbers in DKO Diaphragms

**Figure 3:**
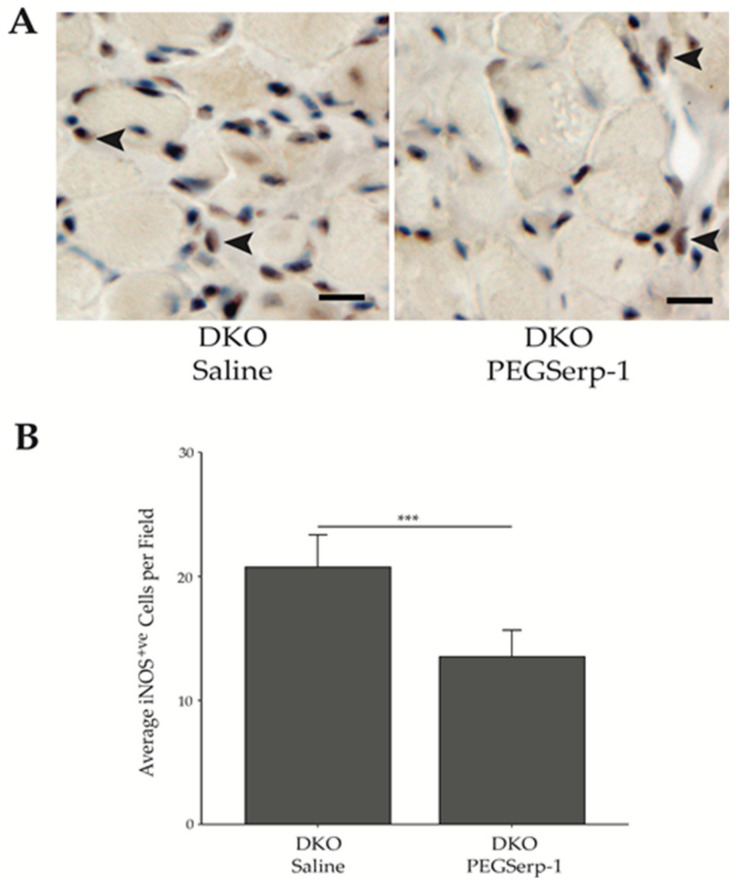
PEGSerp-1 regulation of iNOS+ve M1 macrophage infiltration. (**A**) Representative images of iNOS+ve M1 macrophages in the diaphragm of DKO mice treated with PEGSerp-1 or saline. IHC was performed using an anti-iNOS antibody and the proteins were visualized with DAB and counterstained with hematoxylin (see arrowheads). Scale bars = 10 μm. (**B**) iNOS+ve cells were counted in PEGSerp-1- and saline-treated DKO diaphragms. Data are presented as average number of positive cells per field ± s.d., *** *p* < 0.0001.

In DMD patients, an increase in sarcolemmal membrane instability leads to elevated infiltration of pro-inflammatory M1 macrophages associated with chronic inflammation and cytotoxicity. In an alveoli injury model, Serp-1-induced reduction in inflammation was associated with reduced macrophage invasion [43,44]. Therefore, we examined the impact of the Myxoma virus Serp-1 on M1 macrophage infiltration. IHC was performed using a polyclonal antibody specific for iNOS, a M1 macrophage marker. Diaphragms from DKO mice treated with PEGSerp-1 had an average of 13.52 ± 2.14 s.d. iNOS positive (iNOS+ve) cells per field. This was significantly less than saline-treated control DKO mice that had 20.75 ± 2.61s.d. iNOS+ve cells (Figure 3). This suggests that PEGSerp-1 treatment can reduce the migration of pro-inflammatory macrophages in the chronically injured skeletal muscle environment.

### 3.4. PEGSerp-1 Treatment Does Not Affect M2a Macrophage NUMBERS or Polarization

**Figure 4:**
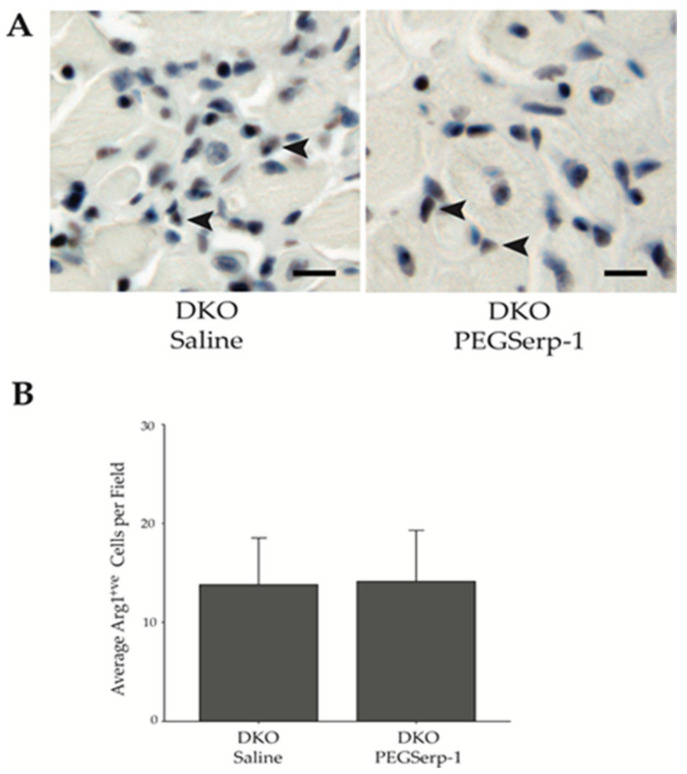
PEGSerp-1 does not alter Arg1+ve M2a macrophage infiltration. (**A**) Representative images of Arg1+ve M2a macrophage in the diaphragm of DKO mice treated with PEGSerp-1 or saline. IHC was performed to detect Arg1+ve single cells, arrowheads indicate example single cells. Scale bars = 10 μm. (**B**) The average number of Arg1+ve cells/field was not significantly different (*p* = 0.84) when comparing PEGSerp-1- and saline-treated DKO mice. Data are presented as average positive cells per field ± s.d.

In response to acute muscle injury, Th2 T cells secrete anti-inflammatory cytokines that induce polarization of alternatively activated, or anti-inflammatory, M2a macrophages, which promote myoblast differentiation [7]. We assessed whether PEGSerp-1 treatment would alter the infiltration or polarization of M2a macrophages in DKO skeletal muscle. As M2a macrophages express arginase (Arg1) [50], we carried out IHC on diaphragms from PEGSerp-1- and saline-treated DKO mice to assess the number of cells using an anti-Arg1 rabbit monoclonal antibody, and the proteins were visualized with DAB. Our data demonstrate that PEGSerp-1-treated DKO muscle had an average of 14.12 ± 5.18 s.d. Arg1 positive (Arg1+ve) cells per field. This was not significantly different than saline-treated DKO muscle, which had an average of 13.80 ± 4.70 s.d. Arg1+ve cells per field, *p* = 0.84 (Figure 4). These data indicate that PEGSerp-1 treatment had no effect on M2a macrophage infiltration or polarization.

### 3.5. PEGSerp-1 Treatment Does Not Alter T Cell Infiltration

T cell infiltration into the damaged muscle is integral for the promotion of both the pro- and anti-inflammatory environments. In mdx mice, both CD4+ and CD8+ T cells are associated with muscle pathology [51]. Thus, we assessed whether treatment with PEGSerp-1 would alter the number, or balance, of T cell types in DKO skeletal muscle. To determine the total number of T cells present, IHC was conducted using a rabbit polyclonal antibody that recognizes the pan T cell marker CD3. There was no significant difference between diaphragms treated with PEGSerp-1, which had an average of 7.43 ± 3.45 s.d. T cells per field, and saline-treated DKO muscle, with an average of 7.82 ± 3.93 s.d. cells per field *p* = 0. 76. To determine whether there was a difference in CD8+ve T cells, IHC was conducted using an anti-CD8 antibody. There was no significant change in the average number of CD8+ T cells in PEGSerp-1-treated, as compared to the saline-treated, DKO diaphragms, which had 8.55 ± 5.08 s.d. per field and 9.11 ± 3.77 s.d. per field, respectively, *p* = 0.70.

## 4. Discussion

**Figure 5:**
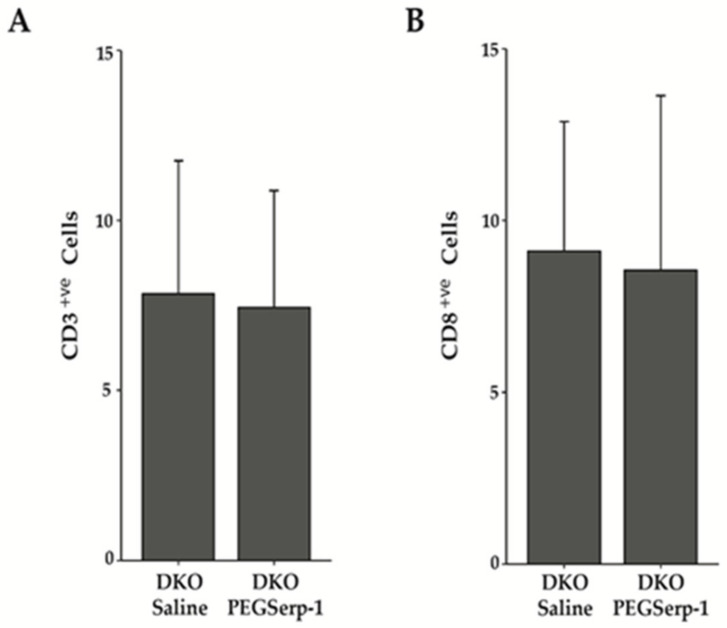
PEGSerp-1 does not alter CD3+ve or CD8+ve T cell infiltration. The presence of CD3+ve and CD8+ve T cells was assessed in the diaphragms of DKO mice treated with PEGSerp-1 and saline using IHC with an anti-CD3 antibody or anti-CD8 antibody. (**A**) The average number of CD3+ve T cells in PEGSerp-1- and saline-treated DKO diaphragms was determined. (**B**) The average number of CD8+ve T cells in PEGSerp-1- and saline-treated diaphragms was determined. Data are presented as average positive cells per field ± s.d., *p* = 0. 70.

Duchenne muscular dystrophy is a genetic condition characterized by a progressive loss of skeletal muscle function due to sarcolemma instability resulting from a deficiency in dystrophin. Reduced efficiency in skeletal muscle contraction and increased endomysial fibrosis are both linked to chronic inflammation caused by repeated microtears [3,4]. We examined whether a pegylated version of the Myxoma virus serpin, Serp-1, would ameliorate the chronic inflammatory environment in *DMD^mdx^/Utrn^−/−^* mice. This protein has been shown to induce an anti-inflammatory response in wound healing, transplants, and other acute injuries without any demonstrated increase in adverse effects in multiple animal models and in one Phase IIa clinical trial [39,40,41,42,43,44,45,52,53]. Systemic PEGSerp-1 treatment of DKO mice significantly decreased muscle fibrosis and the number of infiltrating M1 pro-inflammatory macrophages. There was also a corresponding significant increase in fiber diameter (Figure 1, Figure 2 and Figure 3). We found that PEGSerp-1 treatment did not affect the numbers of M2a Arg1+ve cells, nor T cells, in the muscle (Figure 4 and Figure 5). While Serp-1 treatment has been examined in a wide range of animal models of acute inflammation-induced disease, this is the first study of Serp-1, and specifically PEGSerp-1, as a treatment in a genetic disorder with chronic inflammation as a hallmark symptom. Our data suggest that PEGSerp-1 may be an effective anti-inflammatory in DMD, helping to inhibit the profibrotic environment that contributes to disease progression. Mice were treated with PEGSerp-1 beginning at 4 weeks old, concomitant with symptom onset in this mouse model of DMD [46,47]; however, this indicates that muscle damage has occurred already, and future studies will need to use an alternative delivery method of the Serp-1 protein at younger ages in order to truly determine the long-term outcomes and efficacy. This study was focused on skeletal muscle; therefore, future investigations will be necessary to understand whether PEGSerp-1 can limit fibrosis in cardiac muscle in DMD.

### 4.1. Current Treatment of DMD

Treatment for DMD currently falls into two categories, those trying to target the primary genetic defect or those that target the pathology. The current standard of care for DMD symptoms is immunosuppression with corticosteroids. These drugs bind to the glucocorticoid receptor (GR), which then suppresses pro-inflammatory NF-kB signaling; this is the mechanism of the potent anti-inflammatory drugs prednisolone, prednisone, and deflazacort [31,51]. These glucocorticoids will prolong ambulation and reduce inflammation, but there are many adverse side effects with their long-term use, including weight gain, diabetes, osteonecrosis, hypertension, behavior changes, growth retardation, and muscle weakness and atrophy [31]. Since these drugs prolong ambulation for a limited time only, and have severe adverse effects, there has been development in other pharmacological approaches. Treatment with this immune-modulating serpin, PEGSerp-1, may thus provide a steroid-sparing treatment approach for DMD.

Nonsense mutations are the cause of 10% of DMD; ataluren and gentamycin, a small molecule and an antibiotic, respectively, suppress stop codons during translation, resulting in readthrough and allowing the expression of dystrophin [54,55]. Gentamycin causes irreversible ototoxicity, whereas ataluren is well tolerated, but only slows loss of ambulation for a limited time. Exon skipping can be used to remove exons around the genetic lesion and restore most of the dystrophin protein expression. Dystrophin exon 51 is the target of eteplirsen, the only FDA-approved exon-skipping drug; however, there are other drugs designed to target different dystrophin exons in development currently. These drugs are expensive, require repeated administration, and, while they seem well tolerated, their long-term effects are not known [56]. Therefore, new strategies for immunosuppression with anti-fibrosis functions are essential.

### 4.2. SERP-1

The thrombotic and thrombolytic cascades of serine proteases in the circulating blood and in most tissues, both regulate clot formation after injury, as well as demonstrate bidirectional activation of immune responses. These pathways are regulated by serine protease inhibitors, termed serpins. While tPA and uPA both activate thrombolysis, the uPA/uPAR receptor is now recognized as a central mediator of inflammatory responses, while tPA is considered the key activator of fibrinolysis. Native, unmodified Serp-1, as well as PEGSerp-1, are able to bind and inhibit serine proteases, uPA, tPA, and plasmin, similar to the mammalian serpin, PAI-1. From studies where uPAR has been inactivated, Serp-1 activity, and its ability to promote wound closure and collagen remodeling, is disrupted [52,57,58]. For example, the anti-inflammatory activity of Serp-1 is absent in aortic allograft transplants in uPAR-deficient mice [39]. Furthermore, the efficacy of Serp-1 when used as a topical treatment in full-thickness dermal wound models was blocked by an anti-uPAR antibody [57]. Thus, both studies demonstrate that Serp-1 activity is dependent, in part, on uPAR. Serp-1 treatment reduced intimal hyperplasia in PAI-1-deficient aortic implants. Thus, targeting the uPA/uPAR complex with Serp-1, and investigating all associated interacting component receptors in the inflammatory response, has excellent potential for developing new therapeutics. The native Serp-1 protein has been tested and found safe and effective in animal models of inflammatory vascular disorders, transplants, wound healing, and after spinal cord crush injury [39,40,41,42]. In humans, Serp-1 treatment following coronary artery stent implant in a Phase 2a clinical trial demonstrated reduced markers of heart damage, no side effects (MACE = 0), and no neutralizing antibodies at µg/kg doses in patients. In this trial, Serp-1 was administered immediately after coronary stent implant in patients with unstable coronary syndromes, unstable angina, and non-ST elevation myocardial infarctions, where acute chronic plaque inflammation is seen [53].

The present studies are the first analysis of this virus-derived class of immune-modulating serpin treatment in a genetic muscular dystrophy model, the *DMD^mdx^/Utrn^−/−^* mouse model. PEGSerp-1 treatment resulted in a significant reduction in the number of pro-inflammatory M1 macrophages migrating to the site of the necrotic muscle fibers. This is consistent with the observation that the uPA/uPAR complex regulates the primary invasion of pro-inflammatory macrophages to the site of injury. There was an overall amelioration in the pathology of DMD, as judged by a reduction in fibrosis and an increase in skeletal muscle fiber diameter. PEGSerp-1 modulation of the M1 macrophages is consistent with what has been reported for atherosclerosis, transplant, wound healing, and spinal cord injury [39,40,41,42]. However, an increase in the anti-inflammatory M2 macrophages described in wound healing [52] was not observed in the *DMD^mdx^/Utrn^−/−^* mouse diaphragm. This implies that PEGSerp-1 may not be acting via the same mechanism in skeletal muscle.

Our studies reveal the potential of PEGSerp-1 as a treatment for DMD patients. However, the therapeutic value of PEGSerp-1 may be extended to other inflammatory dystrophies and myopathies treated with corticosteroids [59]. Mutations in dysferlin (dysferlinopathies), which cause progressive Miyoshi myopathy and limb girdle dystrophy type 2B, promote the infiltration of pro-inflammatory immune effector cells comprised mainly of macrophages. Other target conditions could include idiopathic inflammatory myositis [53] and myopathies due to mutations in fukutin and POMT2 [60,61].

There are many differing serpins naturally occurring in mammals and humans that may be associated with chronic inflammation, such as is found in DMD. Serpins also function as inhibitors for activated proteases, and thus may have directed inhibitory actions towards sites where these serine protease pathways are upregulated. More work is necessary to better define mammalian serpin pathways in DMD as potential therapeutic targets. Long-term effects on the development of cardiomyopathies in this DMD model will also be of great interest, both when using PEGSerp-1, or mammalian serpins, as new approaches to treatment.

## 5. Patent

US provisional patent application that has been supplemented and filed internationally via the Patent Cooperation Treaty (PCT) (Provisional patent -63/017,598; Skysong: M20-233L-PR1-f, WSGR ref: 58709-701.101) in the USPTO (04/29/2022).

## Data Availability

All data are contained in the paper.

## Supplementary Materials

The following supporting information can be downloaded at: https://www.mdpi.com/article/10.3390/biomedicines10051154/s1, Figure S1: Distribution of Ferret’s minimal diameters of skeletal muscle fibers after Serp-1 treatment.

## References

[1] Mah J.K., Korngut L., Dykeman J., Day L., Pringsheim T., Jette N. (2014). A Systematic Review and Meta-Analysis on the Epidemiology of Duchenne and Becker Muscular Dystrophy. Neuromuscul. Disord..

[2] Shirokova N., Niggli E. (2013). Cardiac Phenotype of Duchenne Muscular Dystrophy: Insights from Cellular Studies. J. Mol. Cell. Cardiol..

[3] Mavrogeni S., Papavasiliou A., Spargias K., Constandoulakis P., Papadopoulos G., Karanasios E., Georgakopoulos D., Kolovou G., Demerouti E., Polymeros S. (2010). Myocardial Inflammation in Duchenne Muscular Dystrophy as a Precipitating Factor for Heart Failure: A Prospective Study. BMC Neurol..

[4] Klingler W., Jurkat-Rott K., Lehmann-Horn F., Schleip R. (2012). The Role of Fibrosis in Duchenne Muscular Dystrophy. Acta Myol..

[5] Blake D.J., Weir A., Newey S.E., Davies K.E. (2002). Function and Genetics of Dystrophin and Dystrophin-Related Proteins in Muscle. Physiol. Rev..

[6] Arnold H.H., Braun T. (2003). Targeted Inactivation of Myogenic Factor Genes Reveals Their Role during Mouse Myogenesis: A Review. Int. J. Dev. Biol..

[7] Tidball J.G., Villalta S.A. (2010). Regulatory Interactions between Muscle and the Immune System during Muscle Regeneration. Am. J. Physiol. Regul. Integr. Comp. Physiol..

[8] Collins R.A., Grounds M.D. (2001). The Role of Tumor Necrosis Factor-Alpha (TNF-α) in Skeletal Muscle Regeneration: Studies in TNF-α(-/-) and TNF-α(-/-)/LT-α(-/-) Mice. J. Histochem. Cytochem..

[9] Cheng M., Nguyen M.-H., Fantuzzi G., Koh T.J. (2008). Endogenous Interferon-γ Is Required for Efficient Skeletal Muscle Regeneration. Am. J. Physiol. Cell Physiol..

[10] Arnold L., Henry A., Poron F., Baba-Amer Y., van Rooijen N., Plonquet A., Gherardi R.K., Chazaud B. (2007). Inflammatory Monocytes Recruited after Skeletal Muscle Injury Switch into Antiinflammatory Macrophages to Support Myogenesis. J. Exp. Med..

[11] Villalta S.A., Rinaldi C., Deng B., Liu G., Fedor B., Tidball J.G. (2011). Interleukin-10 Reduces the Pathology of Mdx Muscular Dystrophy by Deactivating M1 Macrophages and Modulating Macrophage Phenotype. Hum. Mol. Genet..

[12] Rybakova I.N., Patel J.R., Ervasti J.M. (2000). The Dystrophin Complex Forms a Mechanically Strong Link between the Sarcolemma and Costameric Actin. J. Cell Biol..

[13] Serrano A.L., Muñoz-Cánoves P. (2010). Regulation and Dysregulation of Fibrosis in Skeletal Muscle. Exp. Cell Res..

[14] Saclier M., Cuvellier S., Magnan M., Mounier R., Chazaud B. (2013). Monocyte/Macrophage Interactions with Myogenic Precursor Cells during Skeletal Muscle Regeneration. FEBS J..

[15] Dort J., Fabre P., Molina T., Dumont N.A. (2019). Macrophages Are Key Regulators of Stem Cells during Skeletal Muscle Regeneration and Diseases. Stem Cells Int..

[16] Braga T.T., Agudelo J.S.H., Camara N.O.S. (2015). Macrophages During the Fibrotic Process:M2 as friend and Foe. Front. Immunol..

[17] Bersini S., Gilardi M., Mora M., Krol S., Arrigoni C., Candrian C., Zanotti S., Moretti M. (2018). Tackling Muscle Fibrosis: From Molecular Mechanisms to next Generation Engineered Models to Predict Drug Delivery. Adv. Drug Deliv. Rev..

[18] Lemos D.R., Babaeijandaghi F., Low M., Chang C.-K., Lee S.T., Fiore D., Zhang R.-H., Natarajan A., Nedospasov S.A., Rossi F.M.V. (2015). Nilotinib Reduces Muscle Fibrosis in Chronic Muscle Injury by Promoting TNF-Mediated Apoptosis of Fibro/Adipogenic Progenitors. Nat. Med..

[19] Suelves M., López-Alemany R., Lluís F., Aniorte G., Serrano E., Parra M., Carmeliet P., Muñoz-Cánoves P. (2002). Plasmin Activity Is Required for Myogenesis in vitro and Skeletal Muscle Regeneration in vivo. Blood.

[20] Suelves M. (2005). The Plasminogen Activation System in Skeletal Muscle Regeneration: Antagonistic Roles of Urokinase-Type Plasminogen Activator (Upa) and Its Inhibitor (PAI-1). Front. Biosci..

[21] Suelves M., Vidal B., Serrano A.L., Tjwa M., Roma J., López-Alemany R., Luttun A., de Lagrán M.M., Díaz M.À., Jardí M. (2007). UPA Deficiency Exacerbates Muscular Dystrophy in MDX Mice. J. Cell Biol..

[22] Rahman F.A., Angus S.A., Stokes K., Karpowicz P., Krause M.P. (2020). Impaired ECM Remodeling and Macrophage Activity Define Necrosis and Regeneration Following Damage in Aged Skeletal Muscle. Int. J. Mol. Sci..

[23] Loskutoff D.J., van Mourik J.A., Erickson L.A., Lawrence D. (1983). Detection of an Unusually Stable Fibrinolytic Inhibitor Produced by Bovine Endothelial Cells. Proc. Natl. Acad. Sci. USA.

[24] Ginsburg D., Zeheb R., Yang A.Y., Rafferty U.M., Andreasen P.A., Nielsen L., Dano K., Lebo R.V., Gelehrter T.D. (1986). CDNA Cloning of Human Plasminogen Activator-Inhibitor from Endothelial Cells. J. Clin. Invest..

[25] Nykjaer A., Conese M., Christensen E.I., Olson D., Cremona O., Gliemann J., Blasi F. (1997). Recycling of the Urokinase Receptor upon Internalization of the UPA:Serpin Complexes. EMBO J..

[26] Degryse B., Orlando S., Resnati M., Rabbani S.A., Blasi F. (2001). Urokinase/Urokinase Receptor and Vitronectin/Avβ3 Integrin Induce Chemotaxis and Cytoskeleton Reorganization through Different Signaling Pathways. Oncogene.

[27] Ardite E., Perdiguero E., Vidal B., Gutarra S., Serrano A.L., Muñoz-Cánoves P. (2012). PAI-1–Regulated MiR-21 Defines a Novel Age-Associated Fibrogenic Pathway in Muscular Dystrophy. J. Cell Biol..

[28] Koh T.J., Bryer S.C., Pucci A.M., Sisson T.H. (2005). Mice Deficient in Plasminogen Activator Inhibitor-1 Have Improved Skeletal Muscle Regeneration. Am. J. Physiol. Cell Physiol..

[29] Lluís F., Roma J., Suelves M., Parra M., Aniorte G., Gallardo E., Illa I., Rodríguez L., Hughes S.M., Carmeliet P. (2001). Urokinase-Dependent Plasminogen Activation Is Required for Efficient Skeletal Muscle Regeneration in Vivo. Blood.

[30] Gloss D., Moxley R.T., Ashwal S., Oskoui M. (2016). Practice Guideline Update Summary: Corticosteroid Treatment of Duchenne Muscular Dystrophy: Report of the Guideline Development Subcommittee of the American Academy of Neurology. Neurology.

[31] Kourakis S., Timpani C.A., Campelj D.G., Hafner P., Gueven N., Fischer D., Rybalka E. (2021). Standard of Care versus New-Wave Corticosteroids in the Treatment of Duchenne Muscular Dystrophy: Can We Do Better?. Orphanet J. Rare Dis..

[32] Dekhuijzen P.N., Gayan-Ramirez G., Bisschop A., de Bock V., Dom R., Bouillon R., Decramer M. (1995). Rat Diaphragm Contractility and Histopathology Are Affected Differently by Low Dose Treatment with Methylprednisolone and Deflazacort. Eur. Respir. J..

[33] Gupta A., Gupta Y. (2013). Glucocorticoid-Induced Myopathy: Pathophysiology, Diagnosis, and Treatment. Indian J. Endocrinol. Metab..

[34] Fappi A., de Neves J.C., Sanches L.N., Massaroto e Silva P.V., Sikusawa G.Y., Brandão T.P.C., Chadi G., Zanoteli E. (2019). Skeletal Muscle Response to Deflazacort, Dexamethasone and Methylprednisolone. Cells.

[35] Markham L.W., Kinnett K., Wong B.L., Woodrow Benson D., Cripe L.H. (2008). Corticosteroid Treatment Retards Development of Ventricular Dysfunction in Duchenne Muscular Dystrophy. Neuromuscul. Disord..

[36] Houde S., Filiatrault M., Fournier A., Dubé J., D’Arcy S., Bérubé D., Brousseau Y., Lapierre G., Vanasse M. (2008). Deflazacort Use in Duchenne Muscular Dystrophy: An 8-Year Follow-Up. Pediatr. Neurol..

[37] Barber B.J., Andrews J.G., Lu Z., West N.A., Meaney F.J., Price E.T., Gray A., Sheehan D.W., Pandya S., Yang M. (2013). Oral Corticosteroids and Onset of Cardiomyopathy in Duchenne Muscular Dystrophy. J. Pediatr..

[38] Kim S., Zhu Y., Romitti P.A., Fox D.J., Sheehan D.W., Valdez R., Matthews D., Barber B.J. (2017). Associations between Timing of Corticosteroid Treatment Initiation and Clinical Outcomes in Duchenne Muscular Dystrophy. Neuromuscul. Disord..

[39] Dai E., Guan H., Liu L., Little S., McFadden G., Vaziri S., Cao H., Ivanova I.A., Bocksch L., Lucas A. (2003). Serp-1, a Viral Anti-Inflammatory Serpin, Regulates Cellular Serine Proteinase and Serpin Responses to Vascular Injury. J. Biol. Chem..

[40] Bot I., von der Thüsen J.H., Donners M.M.P.C., Lucas A., Fekkes M.L., de Jager S.C.A., Kuiper J., Daemen M.J.A.P., van Berkel T.J.C., Heeneman S. (2003). Serine Protease Inhibitor Serp-1 Strongly Impairs Atherosclerotic Lesion Formation and Induces a Stable Plaque Phenotype in ApoE−/− Mice. Circ. Res..

[41] Bédard E.L.R., Jiang J., Arp J., Qian H., Wang H., Guan H., Liu L., Parry N., Kim P., Garcia B. (2006). Prevention of Chronic Renal Allograft Rejection by SERP-1 Protein. Transplantation.

[42] Kwiecien J.M., Dabrowski W., Kwiecien-Delaney B.J., Kwiecien-Delaney C.J., Siwicka-Gieroba D., Yaron J.R., Zhang L., Delaney K.H., Lucas A.R. (2020). Neuroprotective Effect of Subdural Infusion of Serp-1 in Spinal Cord Trauma. Biomedicines.

[43] Guo Q., Yaron J.R., Wallen J.W., Browder K.F., Boyd R., Olson T.L., Burgin M., Ulrich P., Aliskevich E., Schutz L.N. (2021). PEGylated Serp-1 Markedly Reduces Pristane-Induced Experimental Diffuse Alveolar Hemorrhage, Altering UPAR Distribution, and Macrophage Invasion. Front. Cardiovasc. Med..

[44] Zhuang H., Han S., Lu L., Reeves W.H. (2021). Myxomavirus Serpin Alters Macrophage Function and Prevents Diffuse Alveolar Hemorrhage in Pristane-Induced Lupus. Clin. Immunol..

[45] Ju B., Guo O., Benissan-Messan D.Z., Shawver M.H., Chen P., Geng B., Wei S., Yaron J.R., Lucas A.R., Zhu H. (2021). Serp-1 Promotes Corneal Wound Healing by Facilitating Re-Epithelialization and Inhibiting Fibrosis and Angiogenesis. Front. Cardiovasc. Med..

[46] Grady R.M., Teng H., Nichol M.C., Cunningham J.C., Wilkinson R.S., Sanes J.R. (1997). Skeletal and Cardiac Myopathies in Mice Lacking Utrophin and Dystrophin: A Model for Duchenne Muscular Dystrophy. Cell.

[47] Deconinck A.E., Rafael J.A., Skinner J.A., Brown S.C., Potter A.C., Metzinger L., Watt D.J., Dickson J.G., Tinsley J.M., Davies K.E. (1997). Utrophin-Dystrophin-Deficient Mice as a Model for Duchenne Muscular Dystrophy. Cell.

[48] Ryder-Cook A.S., Sicinski P., Thomas K., Davies K.E., Worton R.G., Barnard E.A., Darlison M.G., Barnard P.J. (1988). Localization of the Mdx Mutation within the Mouse Dystrophin Gene. EMBO J..

[49] Murphy S., Ohlendieck K., Travascio F. (2016). The extracellular matrix complexome from skeletal muscle. Composition and Function of the Extracellular Matrix in the Human Body.

[50] Ghosh M., Xu Y., Pearse D.D. (2016). Cyclic AMP Is a Key Regulator of M1 to M2a Phenotypic Conversion of Microglia in the Presence of Th2 Cytokines. J. Neuroinflamm..

[51] Spencer M.J., Montecino-Rodriguez E., Dorshkind K., Tidball J.G. (2001). Helper (CD4(+)) and Cytotoxic (CD8(+)) T Cells Promote the Pathology of Dystrophin-Deficient Muscle. Clin. Immunol..

[52] Zhang L., Yaron J.R., Tafoya A.M., Wallace S.E., Kilbourne J., Haydel S., Rege K., McFadden G., Lucas A.R. (2019). A Virus-Derived Immune Modulating Serpin Accelerates Wound Closure with Improved Collagen Remodeling. J. Clin. Med..

[53] Tardif J.C., L’Allier P.L., Grégoire J., Ibrahim R., McFadden G., Kostuk W., Knudtson M., Labinaz M., Waksman R., Pepine C.J. (2010). A Randomized Controlled, Phase 2 Trial of the Viral Serpin Serp-1 in Patients with Acute Coronary Syndromes Undergoing Percutaneous Coronary Intervention. Circ. Cardiovasc. Interv..

[54] Politano L., Nigro G., Nigro V., Piluso G., Papparella S., Paciello O., Comi L.I. (2003). Gentamicin Administration in Duchenne Patients with Premature Stop Codon. Preliminary Results. Acta Myol..

[55] Kerem E., Konstan M.W., De Boeck K., Accurso F.J., Sermet-Gaudelus I., Wilschanski M., Elborn J.S., Melotti P., Bronsveld I., Fajac I. (2014). A Randomized Placebo-Controlled Trial of Ataluren for the Treatment of Nonsense Mutation Cystic Fibrosis. Lancet Respir. Med..

[56] Suñé-Pou M., Limeres M.J., Moreno-Castro C., Hernández-Munain C., Suñé-Negre J.M., Cuestas M.L., Suñé C. (2020). Innovative Therapeutic and Delivery Approaches Using Nanotechnology to Correct Splicing Defects Underlying Disease. Front. Genet..

[57] Dai E., Viswanathan K., Sun Y.M., Li X., Liu L.Y., Togonu-Bickersteth B., Richardson J., Macaulay C., Nash P., Turner P. (2006). Identification of Myxomaviral Serpin Reactive Site Loop Sequences That Regulate Innate Immune Responses. J. Biol. Chem..

[58] Viswanathan K., Liu L., Vaziri S., Dai E., Richardson J., Togonu-Bickersteth B., Vatsya P., Christov A., Lucas A.R. (2006). Myxoma Viral Serpin, Serp-1, a Unique Interceptor of Coagulation and Innate Immune Pathways. Thromb. Haemost..

[59] McNally E.M., Ly C.T., Rosenmann H., Mitrani Rosenbaum S., Jiang W., Anderson L.V.B., Soffer D., Argov Z. (2000). Splicing Mutation in Dysferlin Produces Limb-Girdle Muscular Dystrophy with Inflammation. Am. J. Med. Genet..

[60] Darin N., Kroksmark A.-K., Åhlander A.-C., Moslemi A.-R., Oldfors A., Tulinius M. (2007). Inflammation and Response to Steroid Treatment in Limb-Girdle Muscular Dystrophy 2I. Eur. J. Paediatr. Neurol..

[61] Biancheri R., Falace A., Tessa A., Pedemonte M., Scapolan S., Cassandrini D., Aiello C., Rossi A., Broda P., Zara F. (2007). POMT2 Gene Mutation in Limb-Girdle Muscular Dystrophy with Inflammatory Changes. Biochem. Biophys. Res. Commun..

