# Supplementary material for: Myxomavirus Serp-1 Protein Ameliorates Inflammation in a Mouse Model of Duchenne Muscular Dystrophy
Source: Biomedicines. 2022 May 17;10(5):1154. doi: 10.3390/biomedicines10051154 (PMC9138346; doi:10.3390/biomedicines10051154)
Supplement: Supplementary file 1 [file biomedicines-10-01154-s001.zip › biomedicines-1670682-supplementary.pdf]

biomedicines

MDPI

Supplemental Figure S1 Legend and Methods

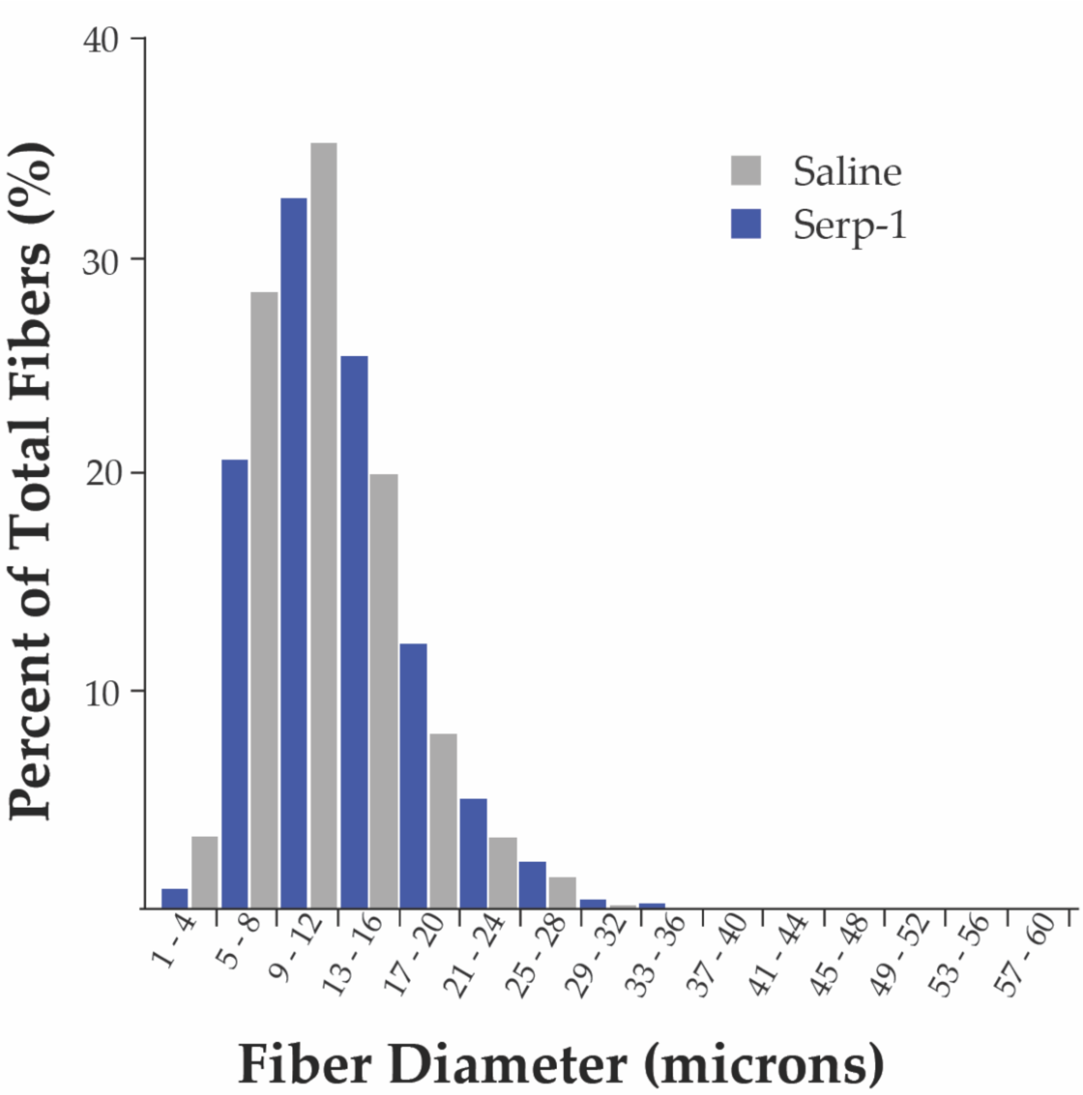

Supplemental Figure S1.

The Ferret's minimal diameter of myofibers was determined for Mason's trichrome stained diaphragms treated with Serp-1 or saline for 28 days. The data is presented as percent of total fibers within 4 µm bins. Treatment with Serp-1 (n=2903 myofibers) resulted in a change in the distribution of fiber size, there was an increase in myofibers with diameters of 13-36 µm, and an overall increased myofiber diameter as compared to saline treated mice (n=3927 myofibers), $p<0.001$.

Methods

After treatment of DKO mice for 28 days with native Serp-1 or saline (section 2.2), the diaphragms were collected, fixed, paraffin embedded, and sectioned at 5 µm. Sections were stained with Mason's trichrome and the Ferret's minimal diameter determined as described in Methods section 2.5.
